# Wave attenuation and trapping in 3D printed cantilever-in-mass metamaterials with spatially correlated variability

**DOI:** 10.1038/s41598-019-41999-0

**Published:** 2019-04-04

**Authors:** Danilo Beli, Adriano T. Fabro, Massimo Ruzzene, José Roberto F. Arruda

**Affiliations:** 1 School of Mechanical Engineering, University of Campinas, Cidade Universitária, Campinas, SP 13083-860 Brazil; 2 Department of Mechanical Engineering, University of Brasilia, Brasilia, DF 70910-900 Brazil; 3 Daniel Guggenhein School of Aerospace Engineering, Georgia Institute of Technology, Atlanta, GA 30332 USA; 4 George W. Woodruff School of Mechanical Engineering, Georgia Institute of Technology, Atlanta, GA 30332 USA

## Abstract

Additive manufacturing has become a fundamental tool to fabricate and experimentally investigate mechanical metamaterials and phononic crystals. However, this manufacturing process produces spatially correlated variability that breaks the translational periodicity, which might compromise the wave propagation performance of metamaterials. We demonstrate that the vibration attenuation profile is strictly related to the spatial profile of the variability, and that there exists an optimal disorder degree below which the attenuation bandwidth widens; for high disorder levels, the band gap mistuning annihilates the overall attenuation. The variability also induces a spatially variant locally resonant band gap that progressively slow down the group velocity until an almost zero value, giving rise to wave trapping effect near the lower band gap boundary. Inspired by this wave trapping phenomenon, a rainbow metamaterial with linear spatial-frequency trapping is also proposed, which have potential applications in energy harvesting, spatial wave filtering and non-destructive evaluation at low frequency. This report provides a deeper understanding of the differences between numerical simulations using nominal designed properties and experimental analysis of metamaterials constructed in 3D printing. These analysis and results may extend to phononic crystals and other periodic systems to investigate their wave and dynamic performance as well as robustness under variability.

## Introduction

Metamaterials and phononic crystals are periodic structures that have been used to control and to manipulate acoustic and elastic waves through band gaps, i.e. frequency bands with no wave propagation^1–5^. Their applications include vibration attenuation^6^ and noise reduction^7^, acoustic cloaking^8^, seismic barriers^9^, acoustic lenses^10^ and topological waveguiding^11,12^. While in phononic crystals the band gap is produced by Bragg scattering due to material or geometric periodic modulation^13^, in metamaterials the band gap effect is created by Mie-type or Fabry-Perot-type resonances due to inclusions that work like local resonators^14,15^. In addition, because of their intrinsicaly complex geometry, 3D printing^16^ has become one of the emerging topics in metamaterials^17–21^. However, manufacturing processes introduce material and geometric variabilities^22,23^, which break the translational periodicity, mistuning the band gaps of the unit cells, and affecting the wave propagation performance in the periodic structure^24,25^. Moreover, this disorder leads to interesting physical phenomena such as energy localization^26–28^, wave trapping (e.g. rainbow metamaterials^29–32^), attenuation bandwidth widening^33–36^ or annihilation^37^.

Even though the effects of correlated disorder in periodic systems and their effect on the Bragg and locally resonant type band gap formation have been widely investigated in other branches of physics^38,39^, very little work has been done on investigating 3D printed structures for structural dynamics applications. In addition, there exists a claim for more experimental investigations in phononic and metamaterial structures manufactured in 3D printing^17–21^. Some differences between numerical simulations and experimental measurements in 3D printed structures have been observed^40–44^, and the variability was pointed out as the cause with no deeper analysis and comprehension of its effect on the wave propagation and dynamic response. Motivated by these previous works, this report investigates numerically and experimentally the physical effects of the spatially correlated variability on the wave propagation of metamaterial beams fabricated using additive manufacturing. Differently from previous works, the metamaterial samples are compared to each other and analyzed from their ensemble statistics.

## Results

**Figure 1:**
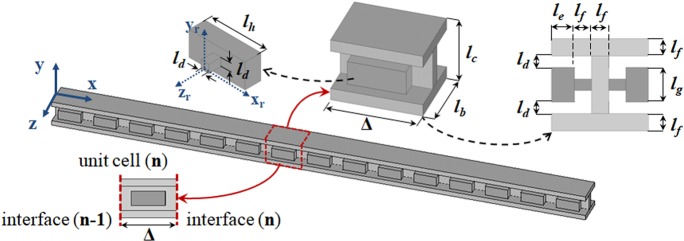
3D view of the cantilever-in-mass metamaterial beam and its unit cell with the nominal geometric dimensions. The unit cell (n) presents an interface (n − 1) at its left boundary and an interface (n) at its right boundary, hence, the metastructure with 15 unit cells has 16 interfaces with ends at interfaces 0 and 15.

In elastic metamaterials, the wave coupling between the host structure and locally resonant inclusions opens the band gap causing the vibration attenuation^45–47^. In this work, the metamaterial model consists of an I-beam with periodically attached cantilever-in-mass resonators on both sides of the web, Fig. 1. The metamaterial beams or metastructures (i.e., finite metamaterials) present 15 unit cells. They are made of polyamide and are fabricated through additive manufacturing using Selective Laser Sintering (SLS). For numerical purposes, the nominal values of the material properties of nylon have been used with elastic modulus ${E}_{0}=1\,{\rm{GPa}}$, mass density ${\rho }_{0}=1000$ kg/m^3^, Poison coefficient ${\nu }_{0}=0.3$, and structural damping $\eta =0.01$. In addition, the nominal geometric dimensions of the unit cell shown in Fig. 1(a) are given by ${\rm{\Delta }}=22\,{\rm{mm}}$, ${l}_{b}=17\,{\rm{mm}}$, ${l}_{c}=16\,{\rm{mm}}$, ${l}_{d}=2\,{\rm{mm}}$, ${l}_{e}=4\,{\rm{mm}}$, ${l}_{f}=3\,{\rm{mm}}$, ${l}_{g}=6\,{\rm{mm}}$, ${l}_{h}=14\,{\rm{mm}}$. The additional mass due to the resonators is around 24% of the I-beam mass.

**Figure 2:**
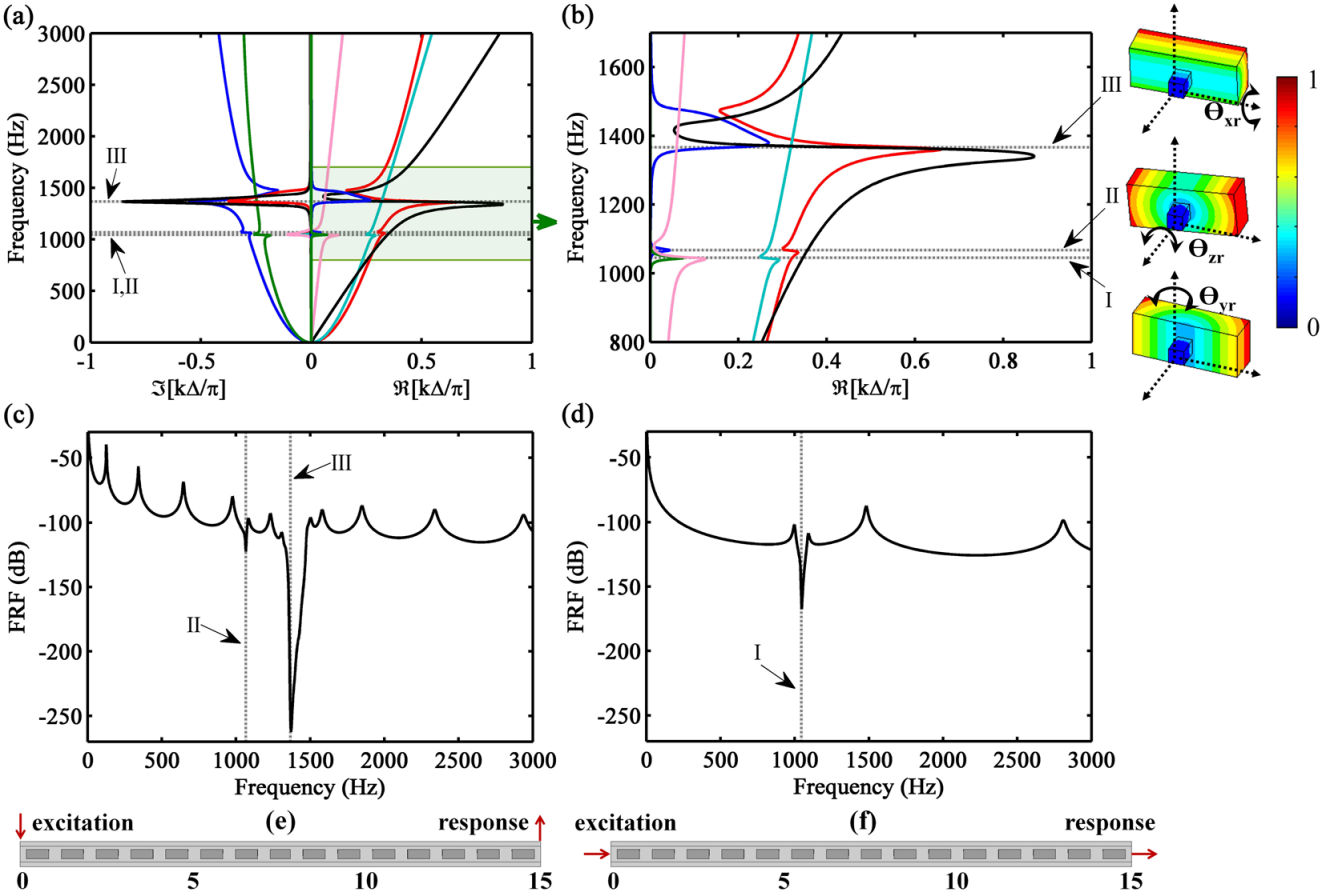
(**a**) Band structure for the metamaterial beam with wave modes related to vertical flexural (red and blue), lateral flexural (cian and green), longitudinal (pink) and torsional (black) motion: propagating component (positive axis) and evanescent component (negative axis) of the wavenumbers. (**b**) Band structure zoom at the band gap zone of $\Re [k{\rm{\Delta }}/\pi ]$ with the natural modes of the cantilever-in-mass resonator responsible for opening the band gaps: I - lateral bending (rotation around y_r_ axis), II - torsional (rotation around z_r_ axis), and III - vertical bending (rotation around x_r_ axis), the colors represent the normalized magnitude of the displacement field. Forced Response Function (FRF) of the metastructure: (**c**) vertical bending motion and (**d**) longitudinal motion. Dynamic response set-up: (**e**) vertical bending motion and (**f**) longitudinal motion, where the force is applied at one end (excitation at interface 0) and the displacement is measured at the other end (response at interface 15) of the metastructure.

The band structure for the unit cell, with six branches, is numerically computed (see Methods) and presented in Fig. 2(a,b). Two of the branches correspond to vertical flexural waves (i.e., the waves are propagating along x direction with displacement field polarized in the y direction), two are due to lateral flexural waves (i.e., the waves are propagating along x direction with displacement field polarized in the z direction), one represents the torsional wave, and one corresponds to the longitudinal wave. The waveshapes that are responsible for the opening of the band gaps corresponding to lateral bending (mode I around 1045 Hz), torsional (mode II around 1065 Hz) and vertical bending (mode III around 1365 Hz) are due to the natural modes of the cantilever-in-mass resonator, shown in Fig. 2(b). Moreover, the torsional mode (i.e., mode III) also opens a small band gap in the vertical flexural waves because the torsion motion of the cantilever-in-mass resonator and vertical bending motion of the structure are coupled. It can be noticed that each wavenumber presents a considerable imaginary part at the local resonances, which means that the corresponding waves propagate, but rapidly decay, thus inducing the vibration attenuation of the dynamic response, Fig. 2(c,d). Besides, the negative derivatives given by the S shapes in the band structure of Fig. 2(a,b) due to locally resonant band gaps can be interpreted as a negative group velocity, i.e., the wave mode propagates energy in the negative direction. This can be viewed as negative dynamic mass or/and stiffness, as a consequence of the assumed time harmonic motion^48,49^. Note that for each propagating bending wave there is a correspondent non-propagating one.

The dynamic responses are shown as displacement Frequency Response Functions (FRF) given by ${G}_{ij}={u}_{j\to i}/{f}_{i}$, where the applied force *f*_*i*_ at point *i* induces a displacement ${u}_{j\to i}$ at point *j*, as in Fig. 2(e,f). In linear systems without time-reversal symmetry breaking (i.e. reciprocal systems), the FRF provides a symmetric response between any two degrees of freedom (DOF), ${G}_{ab}={G}_{ba}$, even for structures with geometrical and material asymmetries, as stated by the Maxwell-Betti theorem^50^. However, in reciprocal systems with any material or geometrical asymmetry, the transmission response ${\tau }_{ij}={u}_{j\to i}/{u}_{i\to i}$ is not symmetrical between two DOFs, ${\tau }_{ab}={G}_{ab}/{G}_{aa}\ne {G}_{ba}/{G}_{bb}={\tau }_{ba}$, which can lead to wrong physical interpretations (e.g., wrongly consider these systems as non-reciprocal).

**Figure 3:**
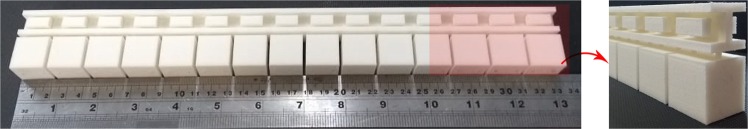
Metamaterial beam with attached cube specimens constructed in SLS 3D printing.

Experimental investigations were performed on a set of ten metamaterial beams constructed from additive manufacturing (see Fig. 3 for a metamaterial sample), which were designed to have the same geometric and material properties in all unit cells. For each metastructure, cubic specimens were printed at each unit cell side such that measurements of the local material properties could be made to estimate the local material properties. This arrangement allowed a link between the dynamic response and material property distribution, and a deeper understanding of the physical phenomena produced by the variability. The experimental set-up (described in Methods) was used for the vertical bending forced response measurements and different vibration attenuation behaviors were observed among the samples (see Supplementary Material). In addition, following the ultrasound experimental set-up (also presented in Methods), the material properties along the metastructure were obtained (see Supplementary Material) and a significant spatial variation was observed.

**Figure 4:**
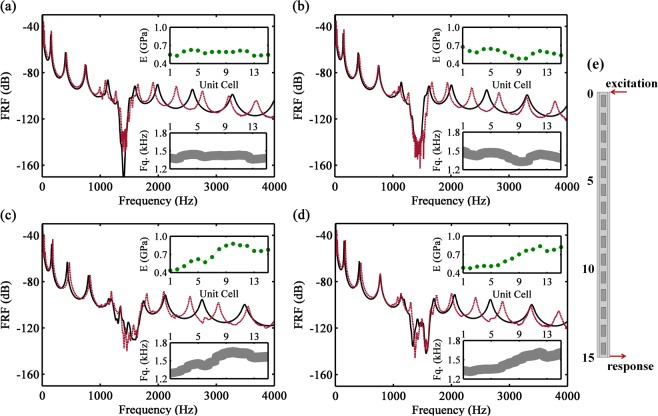
FRF for the metamaterial beam samples: MM1 (**a**), MM2 (**b**), MM3 (**c**) and MM4 (**d**). Legend: experimental measurement in 3D printing model (red), numerical simulation with FE model (black), experimental spatial distribution of elastic modulus (● green) and variation of local band gaps with respect to unit cell location, the colored areas (gray) represent the band gaps. (**e**) Dynamic response set-up for vertical bending measurements.

Firstly, the effects on the band gap due to breaking the periodicity by correlated deterministic variability is investigated by selecting four cases, namely MM1 to MM4. The dynamic responses of the four samples are shown in Fig. 4, where the green lines represent the discrete spatial distribution of the elastic modulus and the gray colored areas are the variation of local band gaps with respect to unit cell location, which can lead to a mismatch of the bands (mistuning). In MM1, a small bandwidth (${\rm{\Delta }}f\approx 250\,{\rm{Hz}}$) with deep attenuation is observed, which is related to a small spatially variation of the material properties. In MM2, the sine shaped spatial distribution produces a slight increase of the attenuation bandwidth (${\rm{\Delta }}f\approx 320\,{\rm{Hz}}$). In MM3, an almost linear variation creates a large bandwidth (${\rm{\Delta }}f\approx 460\,{\rm{Hz}}$) with small attenuation, which can be considered a better attenuation performance when compared to the previous cases. Finally, in MM4, where the variability is similar to MM3, two attenuation regions (${\rm{\Delta }}f\approx 340\,{\rm{Hz}}$) are observed because the spatial distribution of elastic properties is very smooth from cells 1 to 5 and changes rapidly from cell 6 onwards, displaying two distinct levels, one with $E\approx 0.4\,{\rm{GPa}}$ and another with $E\approx 0.7\,{\rm{GPa}}$. Note that cases MM3 and MM4 have approximately the same net change but with different spatial profiles, which strongly influences the attenuation performance. A slowly varying change of the material properties increases the attenuation bandwidth when compared to the rapidly varying change case. By comparing case MM1, with very little variation, with case MM3, the sample with higher variability, the attenuation bandwidth increases more than 80% due to the resonator mistuning. In addition, these experimental results show the high influence of the spatial distribution of material properties on the band gap performance.

The experimental FRFs are also compared to finite element (FE) numerical simulations using the material properties estimated experimentally (the FE model is presented in Methods). It can be noticed that for low frequencies (acoustic branch) and for the vibration attenuation zone, the numerical and experimental results are in a good agreement and the FE model captures the physical behavior involved in the disorder. The FE validation confirms that the spatial distribution of material properties is responsible for the vibration attenuation performance. However, the natural frequencies (peaks in the FRF) do not match for high frequencies (optical branch), which can be attributed to the simplicity of the beam model or to ortrotopy/anisotropy of the material properties. It requires further investigations, beyond the scope of this report.

The resonator variability promotes a mistuning around the fundamental frequency which can lead, as in phononic crystals with gradient index, to Anderson energy localization^51,52^ and wave trapping^44,53^. These phenomena were also observed in quasi-periodic photonic systems that follow the Fibonacci, Thue-Morse, Rudin-Shapiro^54^ sequences or even in systems with random disorder^24,55^ and correlated sequences distributions^29,31^.

**Figure 5:**
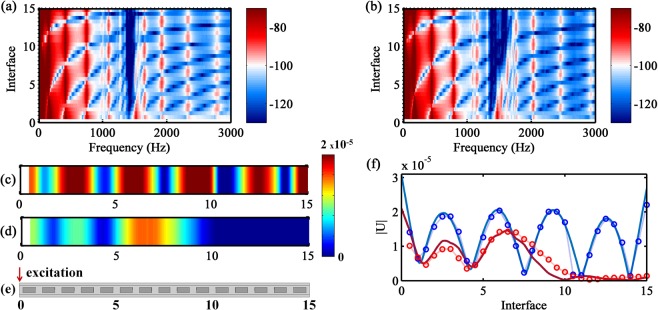
Experimental FRFs as a function of space: (**a**) MM1 and (**b**) MM4, where the colors represent the FRF magnitude in dB. Experimental displacement field: (**c**) MM1 at $f=1654\,{\rm{Hz}}$ and (**d**) MM4 at $f=1632\,{\rm{Hz}}$. (**e**) Dynamic response set-up with excitation location at interface 0. (**f**) Experimental ($\circ$) and FE numerical (–) displacement field: MM1 at $f=1654\,{\rm{Hz}}$ (blue) and MM4 at $f=1632\,{\rm{Hz}}$ (red).

By imposing the excitation force at interface 0 of the metastructure, the experimental FRFs as a function of location for MM1 and MM4 are shown in Fig. 5(a,b). A well defined attenuation in space is achieved for MM1, the vibration localization close to the excitation occurs because the finite nature of the metastructure^36^. However, for MM4, the band gap varies along the metastructure length, which produces a wave trapping phenomenon. This effect appears because, at a fixed excitation frequency for the forward wave propagation, the group velocity progressively slows in space until it reaches an almost zero value^31^. The characteristic S shapes in the band structure of metamaterials present an almost zero group velocity (i.e., ${C}_{g}=2\pi .\{df/d\Re [k]\}=0$), which is produced, in this work, by the natural modes of the cantilever-in-mass resonator at the lower band gap boundary (see Fig. 2(b)). Hence, the lower boundary of these S shapes divides the zones of positive and negative group velocity and supports the wave trapping formation for forward propagation when the band gap varies spatially. The wave trapping zone can also be seen on the band gap boundaries changing as a function of the position along the metastructure, shown in Fig. 4(d). Moreover, a reflection interface is produced at the transition between these two regions, creating a critical section, due to the local change from a propagating to a non-propagating wave, Fig. 5(b). This is also know as a turning point^56^. In Fig. 5(c,d), the displacement fields for the MM1 and MM4 samples are presented for the same vibration mode. In the former, the whole structure vibrates, while in the latter, the wave trapping occurs and concentrates the vibration energy between interfaces 0 and 10, which promotes the attenuation between interfaces 10 and 15. This wave trapping behavior was also validated with FE simulation, Fig. 5(f) (see Supplementary Fig. S2 for the numerical FRFs as a function of space), where a good agreement is achieved between the results.

**Figure 6:**
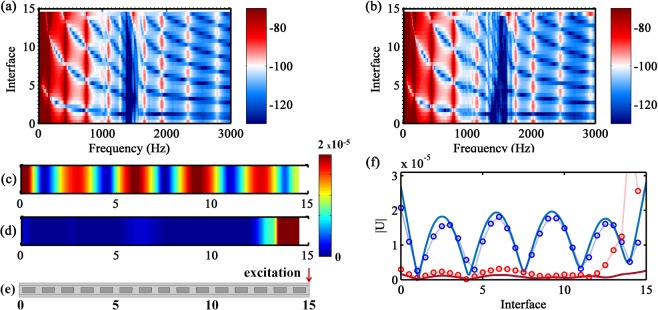
Experimental FRFs as a function of space: (**a**) MM1 and (**b**) MM4, where the colors represent the FRF magnitude in dB. Experimental displacement field: (**c**) MM1 at $f=1654\,{\rm{Hz}}$ and (**d**) MM4 at $f=1632\,{\rm{Hz}}$. (**e**) Dynamic response set-up with excitation location at interface 15. (**f**) Experimental ($\circ$) and FE numerical (–) displacement field: MM1 at $f=1654\,{\rm{Hz}}$ (blue) and MM4 at $f=1632\,{\rm{Hz}}$ (red).

Similar experimental measurements are shown in Fig. 6(a,b) by changing the excitation point to the other metastructure end, i.e., the input force is applied at interface 15. While the displacement field is quite similar for MM1 (Fig. 6(a)), significant differences are observed for MM4, especially at the wave trapping zone, which is not created. Instead, a wider attenuation zone can be noticed (Fig. 6(b)). The backward wave propagation cannot be trapped and only attenuation is observed because the group velocity is not progressively slowing down until it reaches an almost zero group velocity. It is important to notice that the upper band gap boundary is not dominated by the resonator mode, with a well defined zero group velocity, as it happens with the lower band gap boundary. Therefore, the wave is not trapped at this boundary. The FE simulation for the FRFs as a function of space is provided in Supplementary Fig. S2, which is also in agreement with the experimental observations. This behavior is also confirmed by the displacement fields (Fig. 6(c,d)), where the vibration pattern changes with the excitation end inversion for MM4. For MM4, a small difference between experimental and numerical results is observed closer to the excitation point, which can be attributed to the experimental set-up.

In general, the results in Figs 5 and 6 show that the wave trapping effect is related to a broader vibration attenuation bandwidth (MM4), from one end of the metastructure to the other, when compared to the narrower attenuation bandwidth produced by the small material variability sample (MM1). However, for certain applications, the wave trapping can be related to a poor attenuation performance because part of the structure still vibrates and the wave energy is concentrated closer to the reflection interface, which can cause, for instance, structural damage.

The previous results reveal that the vibration attenuation is highly affected by the spatial configuration of the material properties. The attenuation bandwidth increases for a slowly varying, greater net change of the material properties. To better understand the limits of material variations among unit cells to enhance the attenuation bandwidth, FE simulations are performed. For this purpose, a spatially correlated linear variation of the Young’s modulus such that $E(n)={E}_{0}[1+\alpha (n\,{\rm{\Delta }}-L/2)]$ is used, where *n* is the unit cell, *L* is the metastructure length and the slope *α* is the disorder degree.

**Figure 7:**
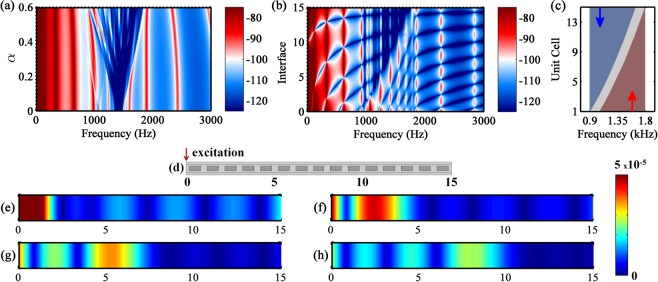
(**a**) FRF parametric analysis of the metastructure with linear distribution of *E*(*n*) (excitation at interface 0 and measurement at interface 15) and (**b**) FRF as a function of space for $\alpha =0.5$ with excitation at interface 0, legend: the colors represent the FRF magnitude in dB. (**c**) Spatially variant band gap for $\alpha =0.5$ (gray colored area) with wave attenuation zone for backward waves (blue colored area) and with wave trapping zone for forward waves (red colored area). (**d**) Dynamic set-up for the trapping of forward waves (i.e., excitation at interface 0) and the correspondent displacement field at: (**e**) 1070 Hz, (**f**) 1244 Hz, (**g**) 1416 Hz and (**h**) 1556 Hz.

The FRFs obtained by changing *α* are shown in Fig. 7(a). The uniform case corresponds to $\alpha =0$ and the resonator mistuning increases with *α*. The attenuation bandwidth is the narrowest for the uniform case ($\alpha =0$ with ${\rm{\Delta }}f\approx 160\,{\rm{Hz}}$) and it reaches a maximum value at $\tilde{\alpha }=0.35$ with ${\rm{\Delta }}f\approx 570\,{\rm{Hz}}$ (i.e., bandwidth widening), which is around 3.5 times larger when compared to the homogeneous case. For $\alpha  > \tilde{\alpha }$, the broadening vibration attenuation disappears because there are individual band gaps at spaced tuned frequencies, which produce vibration peaks between the narrow attenuation zones (i.e., bandwidth annihilation); therefore, the resonator mistuning does not create a continuous broadband attenuation. Finally, it is clear that the attenuation bandwidth increases while the attenuation intensity decreases with the resonator mistuning, and the optimum *α* depends on the amount of resonators. Moreover, for the broadening of the attenuation zone, a smooth variation of the Young’s modulus with a larger number of small resonators is better than a small number of large resonators, as it avoids abrupt changes of the band gap boundaries and the “jagged” profile at the vibration attenuation zone (as seen in Supplementary Fig. S3).

Inspired by the previous wave trapping results, the case with spatially correlated linear variation for $\alpha =0.5$ is analysed. Its FRF as a function of space is shown in Fig. 7(b). For the forward waves (i.e., excitation at interface 0), a clear linear wave trapping-attenuation interface in space and frequency is created, hence the wave penetration length can be linearly controlled^32^. Unlike the rainbow phononic crystals^31^, this phenomenon can be used to tune the wave trapping in space at low frequency and allow spatial wave filtering, non-destructive evaluation as well as energy harvesting, for instance, by placing sensors where the mechanical energy is trapped and at the same time guaranteeing a robust attenuation performance^57^. The spatially variant band gap for $\alpha =0.5$ is shown in Fig. 7(c). For angular frequencies in the interval $[0.900\,1.636]\,{\rm{kHz}}$, the wave trapping is not created by exciting the structure at interface 15 (backward waves - colored blue area). As explained before, only wave attenuation is observed because the group velocity doesn’t gradually slow down until it reaches an almost zero value at the upper band gap boundary (see Supplementary Fig. S3). While for angular frequencies in the range $[1.058\,1.792]\,{\rm{kHz}}$, the wave trapping is created by exciting the structure at interface 0 (forward waves - colored red area) because the group velocity gradually slows down until it reaches a zero value at the lower band gap boundary. For this last case, it is shown that the wave trapping can be localized in specific unit cells by changing the excitation frequency, Fig. 7(e–h), i.e., the structure works as a rainbow metamaterial. Moreover, only vibration attenuation is observed at angular frequencies in the interval $[0.900\,1.058]\,{\rm{kHz}}$ for the excitation of forward waves, and at angular frequencies in the range $[1.636\,1.792]\,{\rm{kHz}}$ for excitation of backward waves, because the interfaces, where excitation is located, are in the band gap.

**Figure 8:**
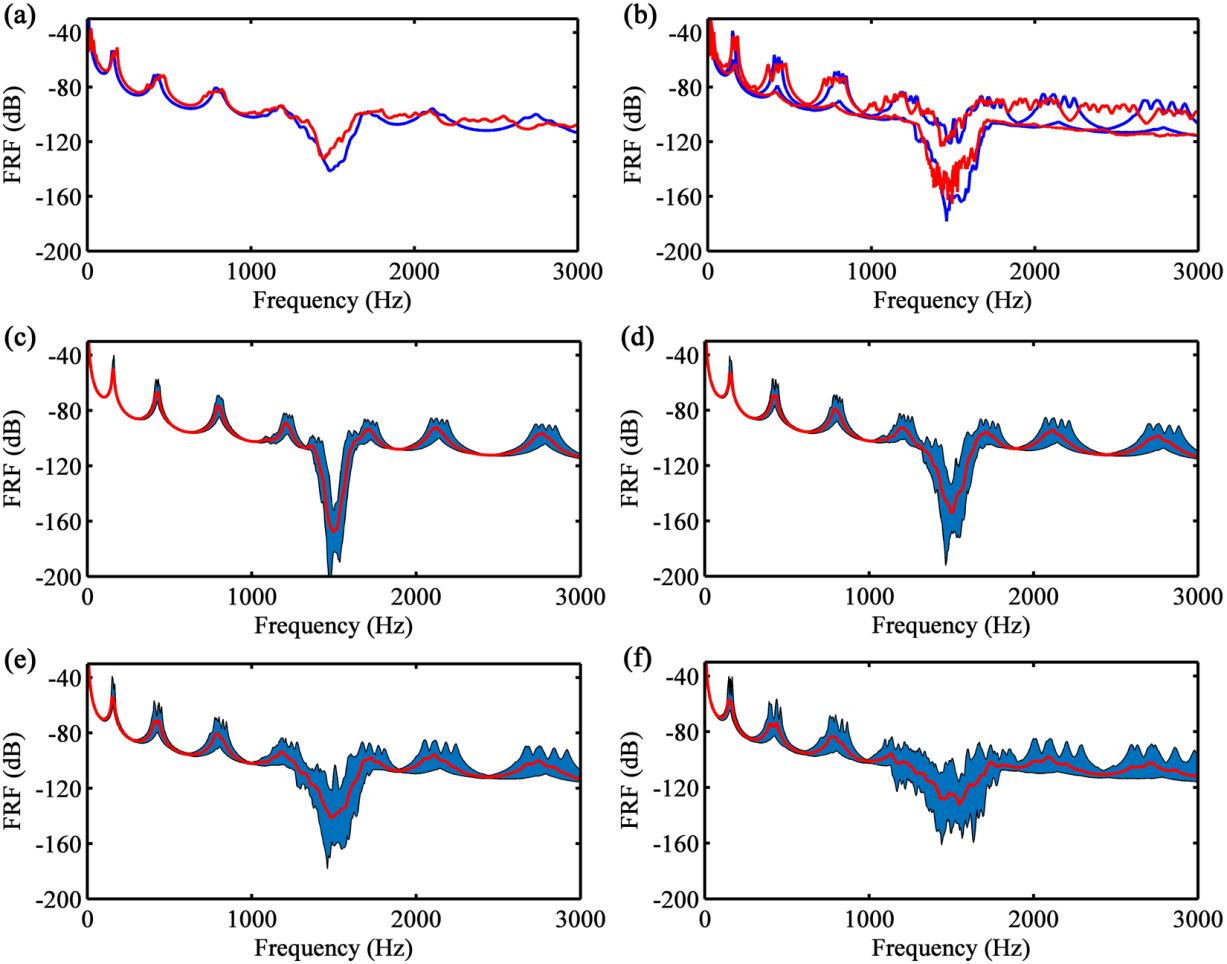
FRF statistics of the metamaterial beam samples obtained by experimental measurements (red) and Monte Carlo simulation (blue): (**a**) mean, (**b**) 5^th^ and 95^th^ percentile. Monte Carlo simulation with 250 samples with: (**c**) $0.50{\sigma }_{\rho ,E}$, (**d**) $0.75{\sigma }_{\rho ,E}$, (**e**) $1.00{\sigma }_{\rho ,E}$ and (**f**) $1.50{\sigma }_{\rho ,E}$: mean (red line), 5^th^ and 95^th^ percentiles (blue colored area).

Finally, the ensemble variability of metastructure samples is considered. The mean, 5^th^ and 95^th^ percentiles of the FRFs of the metamaterial beams obtained from experimental measurements and numerical simulations are shown in Fig. 8(a,b). The numerical validation was performed using the Monte Carlo (MC) simulation with 250 samples. As a first step, the material distributions with spatially correlated random distributions are estimated from a Karhunen-Loeve (KL) expansion, which provides a framework to represent variability and randomness (see Supplementary Material for the theory and numerical results). Secondly, to quantify their effects on the band gap performance, the generated material property samples are included in the FE model to compute the dynamic response. After statistical convergence, the mean as well as the 5^th^ and 95^th^ percentiles of the FRFs are analysed.

A good agreement between the experimental and numerical statistics was achieved, as shown in Fig. 8(a,b). The differences appear mainly at the optical branch frequencies for the reasons discussed before. Moreover, the small divergence inside the vibration attenuation zone can be related to the small number of experimental samples. Variability is observed on the resonance frequencies of the beam as well as on the vibration attenuation zone, which is dominated by the local resonators. Even with large variability of the material properties, which reaches 18% for the elastic modulus (see Supplementary Fig. S1), the vibration attenuation is guaranteed. Therefore, manufacturing metamaterials for vibration attenuation with 3D printers seems feasible because the attenuation zone is robust to the resulting material variability. In the region dominated by the optical branch (after the band gap), the resonance frequencies are highly affected by the variability, producing an almost flat region for the mean and the percentiles. In conclusion, this comparison validates the MC procedure as well as the KL expansion, including the correlation function, correlation length and standard deviation used in the simulations, and can be used to extend the variability limits of the experiment.

The standard deviation of the mass density and the elastic modulus are controlled by the manufacturing tolerances, which are associated to manufacturing time and costs. In general, the smaller the standard deviation, the higher the production costs and the slower the process. However, material variability produces local resonance mistuning that enlarges the attenuation bandwidth, but decreases the attenuation intensity. Therefore, the estimation of the optimum standard deviation or manufacturing tolerances is useful to optimize the production costs and, at the same time, to guarantee the vibration attenuation robustness. In order to predict these optimal values, a stochastic analysis using MC sampling can be performed by changing the material standard deviation employed in the KL expansion. Thus, in Fig. 8(c–f), the MC procedure is applied with fixed correlation length ($b=0.22L$, see Supplementary Fig. S4). The statistics of the dynamic response is shown for ${\sigma }_{\rho ,E}=[0.5,0.75,1.0,1.5]{\tilde{\sigma }}_{\rho ,E}$, where ${\tilde{\sigma }}_{\rho ,E}$ is the measured standard deviation for mass density and elastic modulus of the cube specimens (see Supplementary Material). The local resonance mistuning increases with *σ*, which corresponds to the disorder degree. It can be noticed that the vibration attenuation is guaranteed and enlarged until $\sigma ={\tilde{\sigma }}_{\rho ,E}$, where the larger attenuation bandwidth with the smaller attenuation is observed (i.e., bandwidth widening). For $\sigma  > {\tilde{\sigma }}_{\rho ,E}$, it can be noticed that the metamaterial performance worsen significantly and the resonance tuning fails (i.e., bandwidth annihilation). This is because resonance frequencies can appear between the attenuation zones, similarly to the deterministic linear distributions for $\alpha  > \tilde{\alpha }$. For the case investigated in this work, this analysis shows that the tolerances are within the acceptably limit, and any increase would negatively affect the band gap performance. To the best of the authors knowledge, this stochastic treatment for metastructures was nonexistent or limited in the literature.

## Conclusion

In summary, this report experimentally and numerically investigated the physical effects of spatially correlated variability on the wave propagation performance of 3D printed elastic metamaterials beams. For this purpose, ten metastructures were constructed using an SLS machine, which were analysed individually and as an ensemble. By printing standard cube specimens alongside in each unit cell, the material and geometric properties were spatially tracked, which allowed a link between the dynamic behavior and the variability distribution and, hence, a deeper analysis on the physical phenomena produced by the disorder. The vibration attenuation has been shown to be sensitive to variability even for a band gap at low frequency, and its performance is strictly related to the spatial profile of the variability.

For both deterministic and stochastic approaches, the vibration attenuation bandwidth is broadened with the disorder. However, after an optimal disorder degree, the vibration attenuation bandwidth is annihilated. For the ensemble of metastructures, the optimal disorder that satisfies the attenuation performance and minimizes the production costs was obtained by changing the standard deviation in the MC simulation. In addition, for forward waves, the variability can also induce wave trapping phenomena because of the band gap spatially changing with a group velocity progressively slowing until an almost zero value, which appears at the lower band gap boundary due to resonance of the cantilever-in-mass resonator. However, for backward waves, only attenuation is observed because the group velocity doesn’t slow down until an almost zero value at the upper band gap boundary. This observation was used to propose a rainbow metamaterial with linear space-frequency wave trapping that can be potentially employed for energy harvesting, spatial wave filtering and non-destructive test at low frequency.

Finally, disorder control may be required to avoid undesired wave propagation effects in several applications that are either still at the research level (e.g., mechanical energy of topological wave modes can leak to bulk because of the spatially variant band gap induced by the variability) or have already become industrial applications (e.g., acoustic and vibration barriers may not present the minimum attenuation quality due to implementation and construction variability). The analyses and results presented herein may be applied to other 1D and 2D periodic systems, for instance, to investigate their wave and dynamic robustness as well as performance under disorder.

## Methods

### Numerical simulation

We have implemented the transfer matrix method for the dispersion analysis as well as the dynamic stiffness matrix method for the forced response analysis of the finite system. The equations of motion for the *n*^*th*^ FE unit cell described in frequency domain is ${{\bf{D}}}_{{\bf{c}}{\bf{e}}{\bf{l}}{\bf{l}}}\hat{{\bf{q}}}=\hat{{\bf{f}}}$, where $\hat{{\bf{q}}}$ is the discrete vector of displacements and rotations composed by all degrees of freedom and $\hat{{\bf{f}}}$ is the vector of correspondent external efforts. The dynamic stiffness matrix of the unit cell is given by ${{\bf{D}}}_{{\bf{c}}{\bf{e}}{\bf{l}}{\bf{l}}}={\bf{K}}-{\omega }^{2}{\bf{M}}$, where $\omega$ is the angular frequency, **M** is the mass matrix and **K** is the stiffness matrix. In addition, the material is modeled as linear elastic isotropic, and the structural damping, $\eta$, is modeled as a complex term of the stiffness matrix, **K** = **K**(1 + i*η*). In this work, **M** and **K** matrices are extracted from the software Ansys^®^ and the mesh respects 10 elements per wavelength in order to accurately represents the wave phenomena. Timoshenko beam elements BEAM188, which present two nodes and six degrees of freedom per node, were used to model the one-dimensional metamaterial unit cells.

If no external forces are applied on interior (I) degrees of freedom (DOFs) of the unit cell, ${\hat{{\bf{f}}}}_{{\mathtt{I}}}={\bf{0}}$, these DOFs can be related to the left (L) and right (R) interface DOFs by ${\hat{{\bf{q}}}}_{{\mathtt{I}}}=-\,{{\bf{D}}}_{{\mathtt{II}}}^{-1}({{\bf{D}}}_{{\mathtt{IL}}}{\hat{{\bf{q}}}}_{{\mathtt{L}}}+{{\bf{D}}}_{{\mathtt{IR}}}{\hat{{\bf{q}}}}_{{\mathtt{R}}})$^58^ and a condensed dynamic stiffness matrix (${\tilde{{\bf{D}}}}_{{\mathtt{cell}}}$) is obtained

1 $$[\begin{array}{cc}{\tilde{{\bf{D}}}}_{{\rm{LL}}} & {\tilde{{\bf{D}}}}_{{\rm{LR}}}\\ {\tilde{{\bf{D}}}}_{{\rm{RL}}} & {\tilde{{\bf{D}}}}_{{\rm{RR}}}\end{array}]\,[\begin{array}{c}{\hat{{\bf{q}}}}_{{\rm{L}}}\\ {\hat{{\bf{q}}}}_{{\rm{R}}}\end{array}]=[\begin{array}{c}{\hat{{\bf{f}}}}_{{\rm{L}}}\\ {\hat{{\bf{f}}}}_{{\rm{R}}}\end{array}],$$

where ${\tilde{{\bf{D}}}}_{{\mathtt{BB}}}={{\bf{D}}}_{{\mathtt{BB}}}-{{\bf{D}}}_{{\mathtt{BI}}}{{\bf{D}}}_{{\mathtt{II}}}^{-1}{{\bf{D}}}_{{\mathtt{IB}}}$, with B = [L R]. By assuming the periodic structure free of external forces, the compatibility of displacements as well as the equilibrium of internal forces at the interface of two consecutive unit cells are given by ${\hat{{\bf{q}}}}_{{\mathtt{R}}}^{(n)}={\hat{{\bf{q}}}}_{{\mathtt{L}}}^{(n+1)}$ and ${\hat{{\bf{f}}}}_{{\mathtt{R}}}^{(n)}=-\,{\hat{{\bf{f}}}}_{{\mathtt{L}}}^{(n+1)}$. In addition, the state vector of two consecutive identical interfaces can be related by a transfer matrix which is written in terms of the condensed dynamic stiffness matrix^58,59^ as

2 $$\begin{array}{ccc}{{\bf{u}}}^{(n+1)} & = & {\bf{T}}{{\bf{u}}}^{(n)},\\ {{\rm{w}}{\rm{h}}{\rm{e}}{\rm{r}}{\rm{e}}\,{\bf{T}}} & = & {[\begin{array}{lc}{-{\tilde{{\bf{D}}}}_{{\rm{L}}{\rm{R}}}^{-1}{\tilde{{\bf{D}}}}_{{\rm{L}}{\rm{L}}}} & {{\tilde{{\bf{D}}}}_{{\rm{L}}{\rm{R}}}^{-1}}\\ {-{\tilde{{\bf{D}}}}_{{\rm{R}}{\rm{L}}}+{\tilde{{\bf{D}}}}_{{\rm{R}}{\rm{R}}}{\tilde{{\bf{D}}}}_{{\rm{L}}{\rm{R}}}^{-1}{\tilde{{\bf{D}}}}_{{\rm{L}}{\rm{L}}}} & {-{\tilde{{\bf{D}}}}_{{\rm{R}}{\rm{R}}}{\tilde{{\bf{D}}}}_{{\rm{L}}{\rm{R}}}^{-1}}\end{array}],{{\bf{u}}}^{(n+1)}  =  [\begin{array}{c}{\hat{{\bf{q}}}}_{{\rm{L}}}^{(n+1)}\\{\hat{{\bf{f}}}}_{{\rm{L}}}^{(n+1)}\end{array}]\,{\rm{a}}{\rm{n}}{\rm{d}}\,{{\bf{u}}}^{(n)}=[\begin{array}{c}{\hat{{\bf{q}}}}_{{\rm{L}}}^{(n)}\\ {\hat{{\bf{f}}}}_{{\rm{L}}}^{(n)}\end{array}].}\end{array}$$

Because of the periodicity, the Bloch’s theorem can be applied to relate the state vectors of two consecutive interfaces, ${{\bf{u}}}^{(n+1)}=\lambda {{\bf{u}}}^{(n)}$, where *λ*_*j*_ is the propagation constant ${\lambda }_{j}={e}^{-{\mathtt{i}}{k}_{j}{\rm{\Delta }}}$ and *k*_*j*_ is the wavenumber. Therefore, the eigenvalue problem in Eq. (2) becomes ${\bf{T}}{\varphi }_{j}={\lambda }_{j}{\varphi }_{j}$. The previous eigenproblem provides $2\tilde{n}$ eigensolutions, where $\tilde{n}$ corresponds to the number of DOF associated with each interface. While the eigenvalues (*λ*_*j*_) are associated to phase change or/and attenuation along the structure length, the eigenvectors or wave mode shapes (${\varphi }_{j}$) indicate the spatial distribution of the displacements and internal forces at the interface^58^. Because of the reciprocity, the symmetry in space-time is preserved, and the wave modes appear in pairs (*λ*_*j*_, ${\varphi }_{j}$) and (${\lambda }_{j}^{\ast }$, ${\varphi }_{j}^{\ast }$), which are related to the forward-going ($|{\lambda }_{j}| < 1$) and backward-going ($|{\lambda }_{j}^{\ast }| > 1$) traveling waves^59^.

Finally, the dynamic behavior of finite structures is computed after the assembly of the global dynamic stiffness matrix (**D**_G_) as well as by imposing external forces (**F**_G_) and boundary conditions at the system. The assembly process can be written as ${{\bf{D}}}_{{\mathtt{G}}}={{\mathscr{A}}}^{{\mathtt{N}}}\{{\tilde{{\bf{D}}}}_{{\mathtt{cell}}}\}$, where ${{\mathscr{A}}}^{N}\{\}$ is the finite element-like assembly operator for N unit cells. Therefore, the displacements in the frequency domain (or even the FRFs) are obtained by solving the system of linear equations ${{\bf{U}}}_{{\mathtt{G}}}={{\bf{D}}}_{{\mathtt{G}}}^{-1}{{\bf{F}}}_{{\mathtt{G}}}$.

### Experimental set-up

The experimental variability is investigated by using ten metamaterial beam samples manufactured with polyamide using an SLS machine. Each metamaterial beam sample presents 15 unit cells and a total length of 330 mm. Moreover, standard cube specimens of 20 × 20 × 20 mm were printed on the side of each unit cell to evaluate the material properties along the beam length. Herein, it is assumed that the assembling composed by unit cell and cube specimen has similar material properties and uniform distribution along the unit cell length.

**Figure 9:**
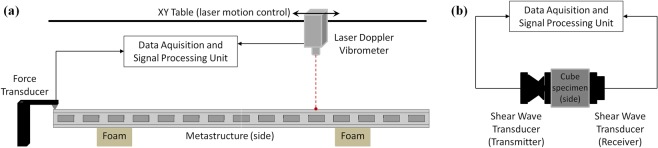
Experimental set-up of the impact test (**a**) and experimental set-up of the ultrasound test (**b**).

The experimental dynamic responses of the metamaterial beams are obtained from impact tests by using a miniature impact hammer with force transducer PCB model 208A02 Piezotronics^®^ to impose an impulse excitation at one end, a Laser Doppler Vibrometer (LDV) from Polytec^®^, which was mounted on an XY table, is used to measure the velocity along the beam, and the LMS SCADAS^®^ system is used for data acquisition and signal processing. See Fig. 9(a) for the experimental set-up of the impact test. A retro-reflective tape is applied to the metastructure surface, enhancing its ability to reflect the incident laser beam. Approximated free-free boundary conditions are imposed by supporting the beam on soft polyurethane foam.

For the experimental material property estimation of the cube specimens, non-destructive ultrasound tests are carried out by using 1 MHz shear wave transducers models U8403072–U84403071 from Olympus^®^ to measure the travel time of longitudinal (*t*_*l*_) and shear (*t*_*s*_) waves, and the Panametrics-NDT™ EPOCH 4 system for data acquisition and signal processing (see Fig. 9(b) for the experimental set-up of the ultrasound test). By assuming isotropic material, the travel times are related to Poisson’s coefficient, elastic modulus and shear modulus by $\nu =[1-2{({V}_{s}/{V}_{l})}^{2}]/[2-2{({V}_{s}/{V}_{l})}^{2}]$, $E=\rho {V}_{l}^{2}(1+\nu )\,(1-2\nu )/(1-\nu )$ and $G=\rho {V}_{s}^{2}$, respectively, where ${V}_{l}={{\rm{\Delta }}}_{x}/{t}_{l}$ and ${V}_{s}={{\rm{\Delta }}}_{x}/{t}_{s}$ are the longitudinal and shear wave speed^60^. The mass density of each cube specimen $\rho$ is computed by $\rho =m/({{\rm{\Delta }}}_{x}{{\rm{\Delta }}}_{y}{{\rm{\Delta }}}_{z})$ in which the mass (*m*) is measured in a precision mass balance while the dimensions $({{\rm{\Delta }}}_{x},{{\rm{\Delta }}}_{y},{{\rm{\Delta }}}_{z})$ are measured with a digital caliper.

## Supplementary information

Supplementary Material
