# Supplementary material for: Wave attenuation and trapping in 3D printed cantilever-in-mass metamaterials with spatially correlated variability
Source: Sci Rep. 2019 Apr 4;9:5617. doi: 10.1038/s41598-019-41999-0 (PMC6449363; doi:10.1038/s41598-019-41999-0)
Supplement: Supplementary file 1 — Supplementary Material [file 41598_2019_41999_MOESM1_ESM.pdf]

Supplementary Material

Wave attenuation and trapping in 3D printed cantilever-in-mass metamaterials with spatially correlated variability

Danilo Beli[1,*], Adriano T. Fabro[2], Massimo Ruzzene[3,4], José Roberto F. Arruda[1]

[1] School of Mechanical Engineering, University of Campinas, Cidade Universitária, Campinas, SP, 13083-860, Brazil
[2] Department of Mechanical Engineering, University of Brasilia, Brasilia, DF, 70910-900, Brazil
[3] Daniel Guggenhein School of Aerospace Engineering, Georgia Institute of Technology, Atlanta, GA, 30332, USA
[4] George W. Woodruff School of Mechanical Engineering, Georgia Institute of Technology, Atlanta, GA, 30332, USA

* Email: dbeli@fem.unicamp.br

1 Material and geometric properties of the cube specimen

By using the experimental set-up presented in Methods, the data that characterize the cube specimens were obtained and shown in Figure S1. Considering all cube specimens of the 10 metastructures, the mean values of the mass density (ρ), elastic modulus (E) and Poisson coefficient (ν) are, respectively, 741 kg/m^3, 0.675 GPa, and 0.267, with standard deviation around 5%, 18.3%, and 6.6%, respectively. The volume of the cubes present variability around 0.8% with mean of 18.3×10^{-6} m^3. Hence, the geometric variability is insignificant with respect to the material property variability, which is the only taken into account for the numerical computations and analysis shown in this work. This fact reveals that the 3D printing in SLS machines is precise for the geometry, but the temperature gradient inside the manufacturing chamber can produce a large variation of the material properties, especially the elastic modulus.

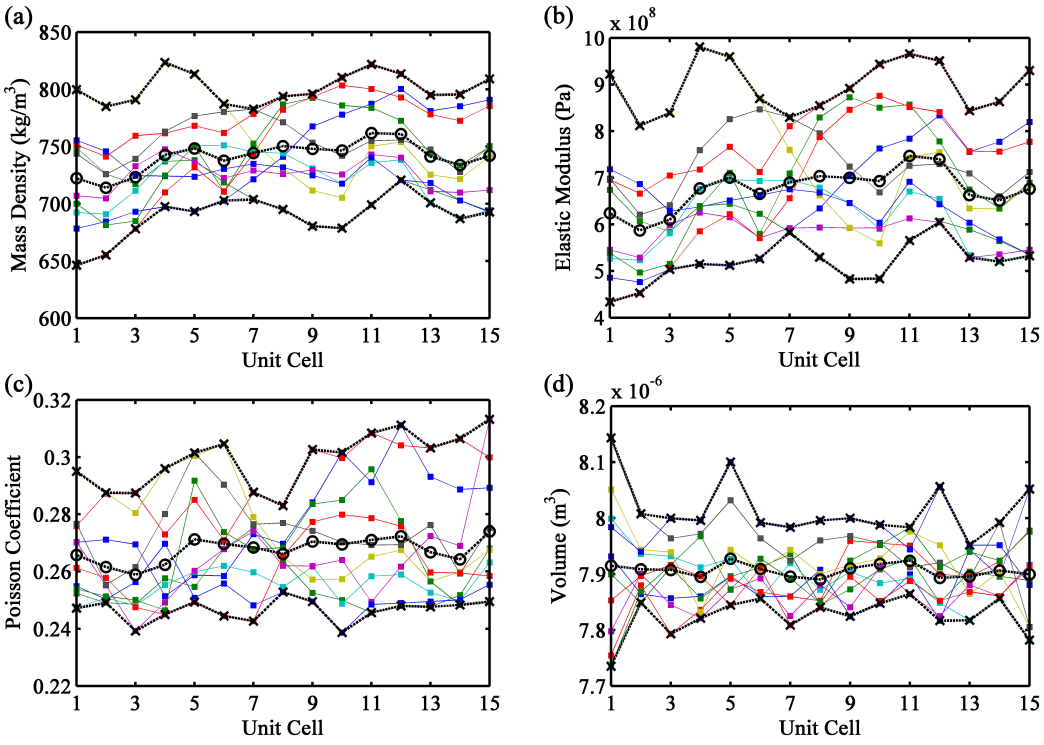

Figure S1. Experimental results for the cubes samples: (a) mass density, (b) elastic modulus, (c) Poisson coefficient and (d) volume. Legend: mean ($-\circ-$), 5th and 95th percentiles ($-$x$-$).

2 FE numerical validation of the wave trapping

The numerical FRF results as a function of space for the samples `MM1` and `MM4`, which were used to validate the experimental wave trapping phenomenon induced by the variability, are shown in Figure S2. In these simulations, the measured material properties were included in the FE model to compute the dynamic response. These numerical results are in agreement with the experimental observations (see Figure 5 and Figure 6), which validates the method for spatially varying material property estimation using the cube specimens. Also, the numerical model confirms that for the `MM4` sample the wave trapping is created for forward wave propagation (i.e., excitation at interface `0`) and only wave attenuation is produced for backward wave propagation (i.e., excitation at interface `15`).

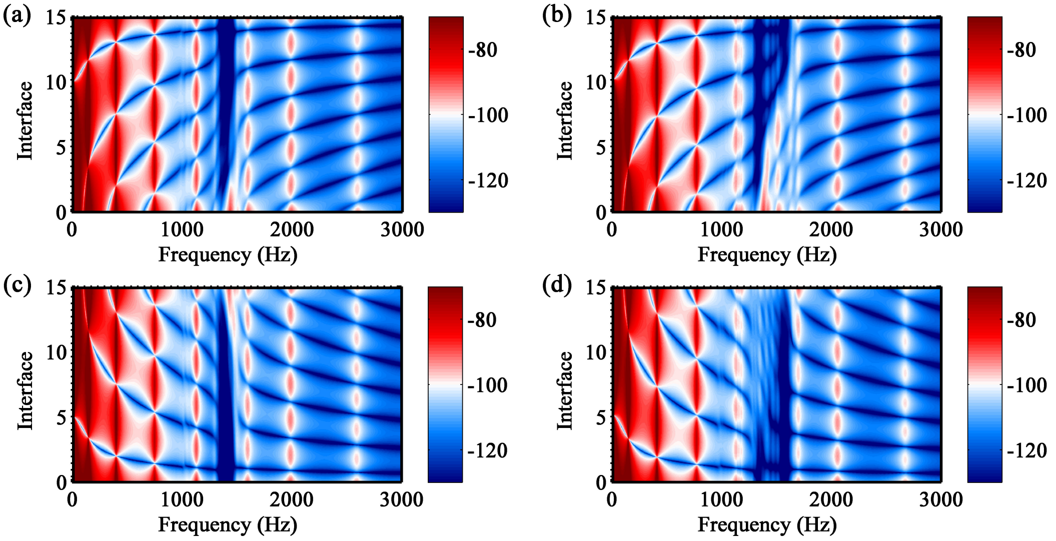

Figure S2. Numerical FRF as a function of space for the samples: `MM1` (a, c) and `MM4` (b, d). Excitation location at interface `0` (a-b) and at interface `15` (c-d). Legend: the colors represent the FRF magnitude in dB.

3 More about the rainbow metamaterial

In this section, more results about the proposed rainbow metamaterial ($E(n) = E_0[1+\alpha(n\ \Delta - L/2)]$ with $\alpha = 0.5$) are presented. The FRF from interface `15` to interface `0` or vice-verse (i.e., $G_{15,0} = u_{0\to15}/f_{15}$, which is equal to $G_{0,15} = u_{15\to0}/f_0$) is presented in Figure S3(a), where the resonance peaks inside the vibration attenuation zone create the "jagged" profile. In Figure S3(b), the FRFs as a function of space are presented for excitation at interface `15`, as discussed in the paper, the wave trapping isn't created even with spatially varying band gap and only wave attenuation is observed because the group velocity doesn't slow down until an almost zero value at the upper band gap boundary. Finally, by exciting the system at interface `15` with the same angular frequencies from Figure 7(e-h), the backward wave propagation isn't trapped at the upper band gap boundary and only vibration attenuation is observed in the displacement fields, Figure S3(d-g).

4 Karhunem-Loeve expansion

A random field $H(x,p)$ can be defined as a collection of random variables indexed by a continuous parameter $x \in D$, where D describes the system domain. In other words, for a given position x_0, $H(x_0,p)$ is a random variable, and for a given outcome p, $H(x,p)$ is a realization of the field. There are several methods available in the literature for generating random fields[S1] and the Karhunem-Loeve (KL) expansion is a special case of series expansion using random variables and deterministic spatial functions, which are orthogonal and derived from the covariance function. A homogeneous random field with a finite, symmetric and positive definite covariance function, defined over a domain is given by[S2]

$$H(x) = H_0 + \sum_{j=1}^{\infty} \sqrt{l_j} X_j f_j(x) \tag{S1}$$

where H_0 is the random field mean value, X_j are random variables, l_j and $f_j(x)$ are eigenvalues and eigenfunctions, solutions of the Fredholm integral equation of the second kind from the covariance function[S2] and the p dependence has been omitted. The

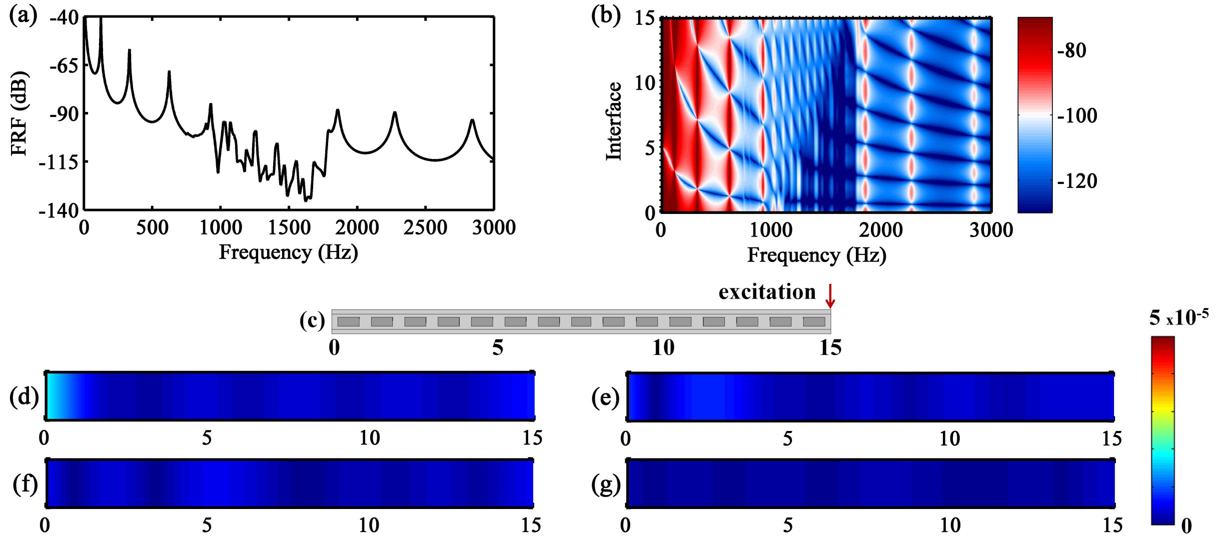

Figure S3. (a) FRF from interface interface 0 to interface 15 ($G_{15,0} = G_{0,15}$). (b) FRF as a function of space and frequency for $\alpha = 0.5$ and excitation at interface 15, legend: the colors represent the FRF magnitude in dB. (c) Dynamic set-up for the excitation of backward waves (i.e., excitation at interface 15) and the correspondent displacement field at: (d) 1070 Hz, (e) 1244 Hz, (f) 1416 Hz and (g) 1556 Hz.

eigenvalues and eigenfunctions can be ordered in descending order of magnitude of the eigenvalues to truncate the series in Equation (S1) to a finite number of terms N_{KL}, chosen by the accuracy of the series in representing the covariance function, rather than the number of random variables[S3]. As a rule of thumb, N_{KL} can be chosen such that $l_j/l_1 < 0.1$, and it depends on the correlation length of the random field. The longer the correlation length the more rapidly the eigenvalues decrease, meaning that fewer terms are needed to accurately represent the series.

If $H(x)$ is a Gaussian random field, X_j are always independent zero mean, unit standard deviation Gaussian random variables. However, if the random field is not Gaussian, it is not possible to use the KL expansion to directly generate $H(x)$ because X_j have unknown joint PDF. Some approaches have been proposed to overcome this issue, amongst them an iterative scheme[S4,S5], which uses directly the KL expansion and can simulate both stationary and non-stationary random fields. Moreover, if the target CDF is approximately Gaussian, only one iteration might be enough to achieve convergence. In general, the eigenproblem can only be solved numerically and normally involves some procedure for discretizing the random field[S1,S6]. However, for some families of correlation functions and specific geometries, there exist analytical solutions of this integral equation[S2].

For the Monte Carlo analysis, the material properties of the unit cell ($\xi = E,\ \rho,\ \nu$) were chosen to be randomly varying according to a random field, i.e., $\xi(x) = H(x)$, where $H(x)$ is a Gamma distributed homogeneous random field with correlation function given by

$$C(\tau) = e^{-|\tau|/b}, \tag{S2}$$

where τ is lag or the distance between any two points in the random field, and b is the correlation length. Then, an analytical solution for Equation (S1) is available[S2]. The properties within each cell are constant, which is usually accurate for large correlation lengths $b > 0.6L$[S7], and given by $H(x_c)$, where x_c is the center of each cell.

The Gamma distribution is chosen according to a Maximum Entropy criterion[S8], which is given by

$$f_\xi(x) = \frac{x^{a_0-1}e^{-x/b_0}}{b_0^{a_0}\Gamma(a_0)}, \quad x > 0, \tag{S3}$$

where $\Gamma(x) = \int_0^{+\infty} t^{x-1}e^{-t}dt$, $x > 0$ is the Gamma function and the parameters of the distribution are given by $a_0 = 1/\delta_\xi^2$ and $b_0 = \xi_0\delta_\xi^2$, where δ_ξ is the dispersion parameter. Equation (S1) is used to simulate the non-Gaussian random field[S4,S5] in which the X_j are firstly generated using Gamma independent random variables and the Latin Hypercube Sampling (LHS) scheme as a stochastic solver[S9].

By knowing the experimental spatial distribution of material properties, Figure S1, the correlation function and length of Equation (S2) can be estimated, which can be used to estimate numerically the material property distributions and, hence, to provide a significant physical insight in the stochastic analysis of the ensemble of metastructures.

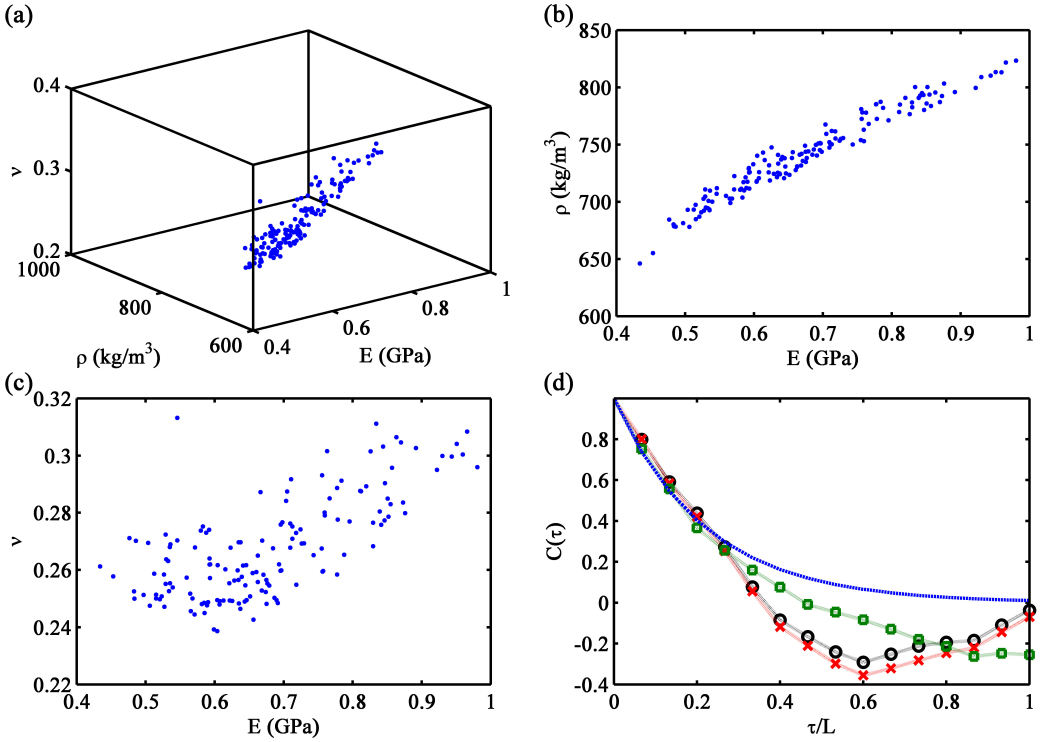

Figure S4. (a) E-ρ-ν 3D scattering plot. (b) E-ρ scattering plot. (c) E-ν scattering plot. (d) Estimation of the correlation length for $C(\tau)$ function: analytic expression (—— blue), experimental E (x red), experimental ρ (○ black) and experimental μ (□ green).

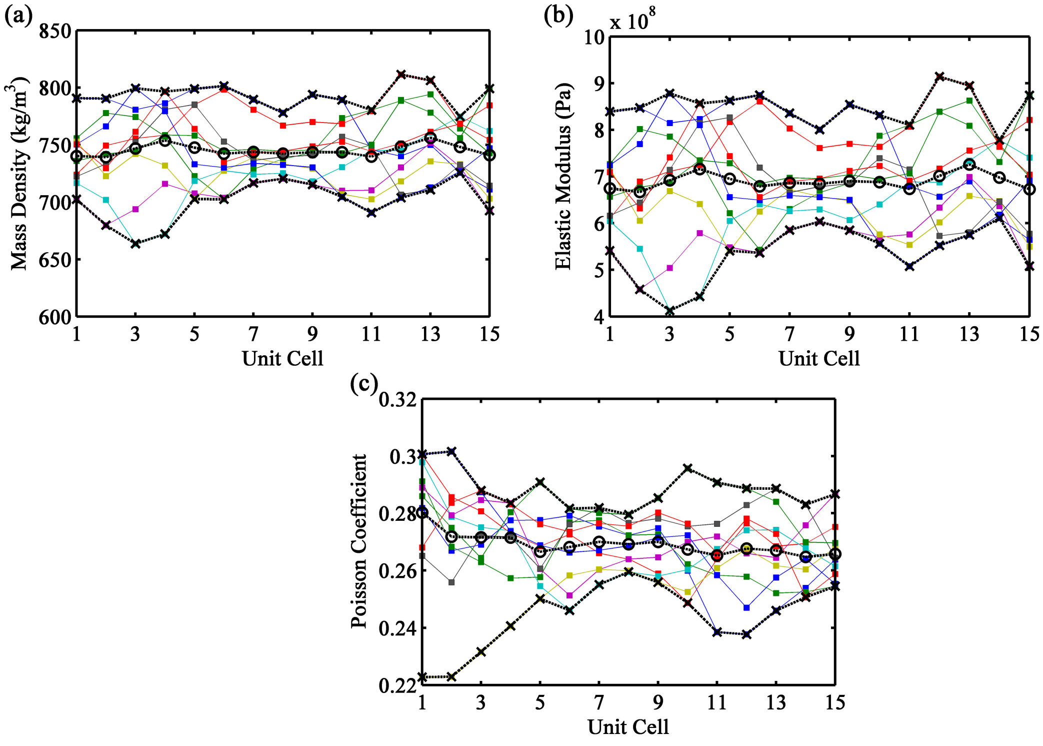

Figure S5. (a) KL estimation for the mass density. (b) KL estimation for the elastic modulus. (c) KL estimation for the Poisson coefficient. Legend: mean (− ○ −), and 5th and 95th percentiles (−x−).

The experimental mass density and elastic modulus are highly correlated, as presented in the scatter plot of Figure S4(a-b). Due to the manufacturing process, they depend on a single parameter, which is the porosity. Moreover, the Poisson coefficient is only slightly correlated with these two parameters, Figure S4(a,c). By using Equation (S2) as a proposed correlation function, the correlation length was estimated as $b = 0.22L$ for all the three material parameters, as seen in Figure S4(d). The experimental correlation was calculated by using an unbiased FFT-based estimator. Because the correlation length is larger than three times the unit cell length ($a/b \approx 3.3$), the material properties inside the unit cell can be considered constant. The correlation function given by Equation (S2) with $b = 0.22L$, and the experimental standard deviations for mass density, elastic modulus and Poisson coefficient were applied on the KL expansion to generate numerical spatial profiles of material properties, and 10 random samples are shown Figure S5.

5 Comparison between experimental and numerical FRFs

Finally, the dynamic response of the metamaterial beam samples are shown in Figure S6 for all the 10 experimental measurements as well as for the FE simulation by using the KL estimation for the 10 material distributions presented in Figure S5. A similar dynamic behavior can be observed, which is also shown in the validation and discussions of Figure 8(a-b), where 250 material distributions were used for the sake of convergence in the Monte Carlo simulation.

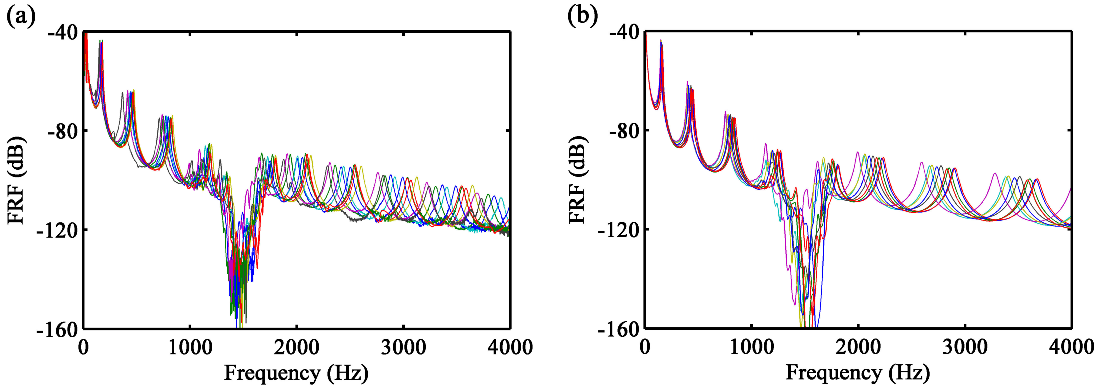

Figure S6. FRFs for the metamaterial beam samples: (a) experimental measurements and (b) FE simulation with KL estimation of the material properties.

References

[S1] Stefanou, G. The stochastic finite element method: Past, present and future. *Comput. Methods Appl. Mech. Eng.* **198**, 1031–1051 (2009). 9–12.

[S2] Ghanem, R. & Spanos, P. D. *Stochastic Finite Elements: A Spectral Approach* (Dover Publications, Minneola, N.Y., 2012), revised edition edn.

[S3] Huang, S. P., Quek, S. T. & Phoon, K. K. Convergence study of the truncated Karhunen–Loeve expansion for simulation of stochastic processes. *Int. J. for Numer. Methods Eng.* **52**, 1029–1043 (2001).

[S4] Phoon, K. K., Huang, S. P. & Quek, S. T. Simulation of second-order processes using Karhunen–Loeve expansion. *Comput. & Struct.* **80**, 1049–1060 (2002).

[S5] Phoon, K. K., Huang, H. W. & Quek, S. T. Simulation of strongly non-Gaussian processes using Karhunen–Loeve expansion. *Probabilistic Eng. Mech.* **20**, 188–198 (2005).

[S6] Betz, W., Papaioannou, I. & Straub, D. Numerical methods for the discretization of random fields by means of the Karhunen–Loève expansion. *Comput. Methods Appl. Mech. Eng.* **271**, 109–129 (2014).

[S7] Li, C. & Der Kiureghian, A. Optimal discretization of random fields. *J. Eng. Mech.* **119**, 1136–1154 (1993).

[S8] Soize, C. *Uncertainty Quantification - An Accelerated Course with Advanced Applications in Computational Engineering*. Interdisciplinary Applied Mathematics (Elsevier, 2017), 1st edn.

[S9] Rubinstein, R. & Kroese, D. *Simulation and the Monte Carlo method.* Wiley Series in Probability and Statistics (John Wiley & Sons, Inc., Hoboken, NJ, USA, 2007), second edition edn.
